# The use of protein supplements in children with cerebral palsy: A scoping literature review

**DOI:** 10.1371/journal.pone.0322730

**Published:** 2025-05-08

**Authors:** Ineke Verreydt, Els Ortibus, Anja Van Campenhout, Kaat Desloovere, Daisy Rymen

**Affiliations:** 1 Department of Rehabilitation Sciences, KU Leuven, Leuven, Belgium; 2 Department of Development and Regeneration, Faculty of Medicine, KU Leuven, Leuven, Belgium; 3 Pediatric Orthopedics, Department of Orthopedics, University Hospitals Leuven, Leuven, Belgium; 4 Clinical Motion Analysis Laboratory, University Hospitals Leuven, Pellenberg, Belgium; IRCCS Medea: Istituto di Ricovero e Cura a Carattere Scientifico Eugenio Medea, ITALY

## Abstract

The aim of this scoping review was to examine the literature regarding the use of protein supplements to improve macroscopic muscle properties in a pediatric population in general, and more specifically in children with cerebral palsy. Based on our prospectively registered protocol (https://doi.org/10.17605/OSF.IO/8DM9G), a systematic literature search was performed in five databases and two clinical registers. Studies were selected by two independent reviewers using predefined selection criteria, and data were summarized using a data extraction form. A broader search on adults with cerebral palsy and the general pediatric population was considered to be relevant due to the limited number of studies conducted in children with cerebral palsy. After deduplication, 5207 records were identified and screened. A total of 18 publications were included in the current review. Two studies were performed in individuals with cerebral palsy, eight in healthy children, two in children with respiratory problems, one in critically ill children, one in children with end-stage liver disease, one in children and adolescents undergoing treatment for a pediatric malignancy, one in children with Pompe disease and two in children with Duchenne muscular dystrophy. The different muscle parameters reported were muscle volume, muscle mass, fat-free mass and fat-free mass index, lean body mass and lean body mass percentage, arm muscle area and muscle cross-sectional area of the arm, thigh and calf. The heterogeneity of the included studies and their moderate quality level made it difficult to draw solid overall conclusions. More research is needed on the use of protein supplements in children with cerebral palsy. However, supplementation with branched-chain amino acids, in particular leucine, might be promising.

## Introduction

With a prevalence of 3.4 per 1000 live births in low- and middle-income countries and 1.5 per 1000 live births in high-income countries, cerebral palsy (CP) is the leading cause of physical disability in children [1,2]. This neurological disorder results from an upper motor neuron lesion in the developing fetal or infant brain [1]. While the brain lesion is static, the related musculoskeletal problems become progressively worse over the years [1,3]. Hampered muscle growth, including reduced muscle volume, forms an important problem in children with CP [4–6]. Although different contributing mechanisms have been proposed, their precise role in altering macroscopic muscle properties remains to be explored [7].

Exercise, especially resistance training, and nutrition have been described as potent stimulators of muscle protein synthesis [8]. A recent intervention study by Hanssen et al. (2022) aimed to improve muscle volume and muscle growth in children with CP. The study investigated the effect of a 12-week progressive resistance training program on muscle morphology, and showed only limited improvements in macroscopic muscle properties [9]. Two recent reviews concluded that the effect of resistance training on macroscopic muscle morphology is still inconclusive [10,11]. In children with CP, an increased incidence of nutritional problems has been reported, which may result in an inadequate protein intake [7]. Anker-van der Wel et al. (2020) investigated the dose, timing and source of protein intake in children with CP and showed sub-optimal protein intake during breakfast and lunch [12]. Since an adequate protein intake throughout the day is required to maintain a positive net protein balance for muscle protein synthesis, protein supplementation at specific time points during the day may be useful to improve macroscopic muscle properties [8]. A wide variety of different protein and individual amino acid supplements are available. For protein supplements, a distinction is often made between whey, casein, and soy protein supplements [13]. Tang et al. (2009) showed that whey hydrolysate or soy protein had a greater effect on muscle protein synthesis than casein. Furthermore, muscle protein synthesis was stimulated to a greater extent after whey hydrolysate than after soy consumption following resistance exercise [13]. In addition, individual amino acid supplements, and more specific branched-chain amino acids (BCAA), have gained interest. In particular, the BCAA leucine seems to play an important role in stimulating muscle protein synthesis and reducing muscle protein breakdown [14].

The benefit of protein supplementation has mainly been studied in athletes, elderly and critically ill patients. Many athletes, professionals as well as recreationalists, consume protein supplements to achieve muscle hypertrophy and to limit the structural muscle damage associated with their training modalities [15,16]. Two recent systematic reviews on the use of protein supplements in healthy adults indicated that protein supplementation significantly promoted gains in muscle size during prolonged resistance training [17]. In the elderly, protein supplementation in addition to resistance training resulted in an increased muscle mass, as stated by the umbrella review by Gielen et al. (2021) [18]. Moreover, the use of the BCAA leucine was mainly beneficial in elderly with sarcopenia [18]. In critically ill patients, muscle wasting was found to be associated with an increased mortality risk [19]. Although protein supplementation has been used to counter muscle loss, interpretation of the results is complicated by the occurrence of feeding-resistant catabolism in the critically ill, and increasing evidence that fasting-activated pathways serve as an adaptive mechanism to cope with severe illness [20,21].

Despite the frequent use of protein supplements to prevent muscle wasting or promote muscle hypertrophy, only few studies have been conducted on the use of protein supplements in a pediatric population and more specific in children with CP. Therefore, we conducted a scoping review to examine the extent, range and nature of the available research and to summarize the literature regarding the state-of-the-art in the use of protein supplementation to increase macroscopic muscle properties. This review specifically focused on muscle volume, in a pediatric population in general, and more specifically in children with CP. The research questions were: (1) What protein supplements are used in a pediatric population, with the main objective of increasing muscle volume? (2) What is known from the existing literature about the benefits and adverse effects of protein supplements on muscle hypertrophy in children with CP?

## Materials and methods

### Study design

This scoping review was conducted to map the relevant literature on the use of protein supplements to improve macroscopic muscle properties, with a main focus on muscle volume, in a pediatric population in general and more specifically in children with CP. The methodology of this scoping review was established based on the methodological framework of Arksey & O’Malley (2005), and the manual for scoping reviews published by the Joanna Briggs Institute (2020) [22–24].

### Protocol and registration

The protocol of this scoping review is based on the Preferred Reporting Items for Systematic Reviews and Meta-analysis Protocols (PRISMA-P), the Preferred Reporting Items for Systematic Reviews and Meta-analysis extension for Scoping Reviews (PRISMA-ScR) and the Best practice guidance and reporting items for the development of scoping review protocols by Peters et al. (2022) [25–27]. The protocol was reviewed by the research team using the PRISMA-P and PRISMA-ScR checklists. The completed PRISMA-P and PRISMA-ScR checklists are presented in the supporting information (S1 and S2 Files, respectively). The final protocol was prospectively registered with the Open Science Framework on 27/02/2023 (https://doi.org/10.17605/OSF.IO/NU42F) and updated on 03/04/2024 (https://doi.org/10.17605/OSF.IO/8DM9G) (S9 File).

### Eligibility criteria

As stated in the updated manual for scoping reviews published by the Joanna Briggs Institute (2020), the Population, Concept and Context (PCC) eligibility criteria were preferred when conducting a scoping review [24]. The PPC eligibility criteria were described as follows:

#### Population.

The main objective was to obtain more insight into the use of protein supplements to increase muscle volume in children with CP. Expanding the search strategy to include children in general, and adults with CP was considered relevant since, to our knowledge, no studies have been conducted on the use of protein supplements to increase muscle volume in children with CP. Specific inclusion criteria were: (1) CP: confirmed diagnosis, no age limitation, (2) Children: age 2–12 years, healthy or diagnosed with a condition other than CP. Studies that included only undernourished or malnourished participants were excluded.

#### Concept.

Specific inclusion criteria were: (1) Intervention: protein or amino acid supplementation, (2) Comparator(s)/control(s): with or without, (3) Primary outcome: macroscopic muscle properties (e.g., volume, cross-sectional area, length), and alternative parameters for muscle properties such as lean body mass, (4) Secondary outcome: adverse effects. Exclusion criteria were: (1) Intervention: parenteral administration, (2) Outcome: microscopic muscle parameters, muscle protein kinetics derived from arterial or venous blood samples. All types of published articles were considered relevant, but case reports and case series were not preferred due to their descriptive nature, and were therefore interpreted with extra caution. Reviews were included if a meta-analysis was performed. If not, the individual relevant papers mentioned in the review were screened and included if they met the inclusion criteria. Only articles written in English were included. No time criteria were considered.

#### Context.

The context of the eligibility criteria was not included in this scoping review due to its irrelevance to the current topic.

### Information sources and search

Comprehensive literature searches of electronic bibliographic databases were conducted by two independent researchers (I.V. and D.R), in Scopus, Web of Science, PubMed, EMBASE and CENTRAL (Cochrane). Pre-defined search terms and MeSH terms were used. Clinicaltrials.gov and ICTRP were searched for registered clinical trials. Titles and abstracts of articles obtained through the structured search were screened based on predefined eligibility criteria, before analyzing the full article. Reference lists of all relevant reviews and included papers were also scanned. The search strategy was developed with the assistance of the biomedical reference librarians of the KU Leuven Libraries – 2Bergen – Learning Centre Désiré Collen (Leuven, Belgium). The search strategy was peer-reviewed by an additional expert (K.D.) using the Peer Review of Electronic Search Strategies (PRESS) checklist and an extension to the PRISMA Statement for reporting literature searches in systematic reviews (PRISMA-S checklist) (S3 and S4 Files) [28,29]. The detailed search strategy used for each database, is presented in the supporting information (S5 File). The search strategy is reported using Extension to the PRISMA Statement for reporting Literature Searches in Systematic Reviews checklist (PRISMA-S) (S4 File) and the PRISMA 2020 flow diagram for new systematic reviews [29,30]. The search was performed on 02/03/2023 and updated on 13/06/2024.

### Selection of sources of evidence, data items and data charting process

Data extraction was performed independently by a pair of researchers (I.V. and D.R.). Each phase of the review (screening, eligibility, inclusion) was preceded by a training session. Titles and abstracts were screened using the a priori defined eligibility criteria. Subsequently, full-text articles and citations were screened for inclusion. Inter-rater discrepancies were resolved by discussion or by a third reviewer (K.D.). Data on article characteristics (e.g., type of article or study, country of origin, year of publication, first author, last author and whether the study was sponsored), participant characteristics (e.g., population or patient group, age category, gender, number of included participants and whether a control group was used), intervention characteristics (e.g., type of supplements used, duration and dosage, timing and form of administration), control characteristics (supplementation), outcome characteristics (e.g., primary outcome parameters, secondary outcome parameters and adverse effects) and result characteristics (e.g., statistical analysis performed yes or no, summary of results of muscle outcomes) were abstracted using a predefined data extraction chart. The standardized data extraction form was developed a priori and pilot-tested among all reviewers. The EndNote Desktop reference manager was used to deduplicate records from multiple database searches and other information sources. A second check for deduplication was performed manually. Screening was conducted by two independent researchers using the online program Rayyan (https://www.rayyan.ai).

### Critical appraisal of individual sources of evidence

According to the recommendations of the manual for scoping reviews published by the Joanna Briggs Institute (2020), an assessment of the methodological quality of the included articles in a scoping review is not required [23,24]. However, to improve critical appraisal of the included studies, the Downs and Black Checklist for Quality Assessment, developed to assess the quality of both randomized controlled and non-controlled trials, was used to assess the quality of all included papers [31]. Due to the nature of this review, the scoring of item 27 was modified (rating was based on whether or not the study performed a power calculation, rather than rating the available range of study powers), resulting in a maximum possible score of 28 instead of 32. In addition, every study was assigned a quality level: excellent (score 26–28); good (score 20–25); fair (score 15–19); and poor (score < / = 14). The scoring was performed by a single reviewer (I.V.), but discussed with the second reviewer (D.R.).

## Results

### Study selection

**Fig 1:**
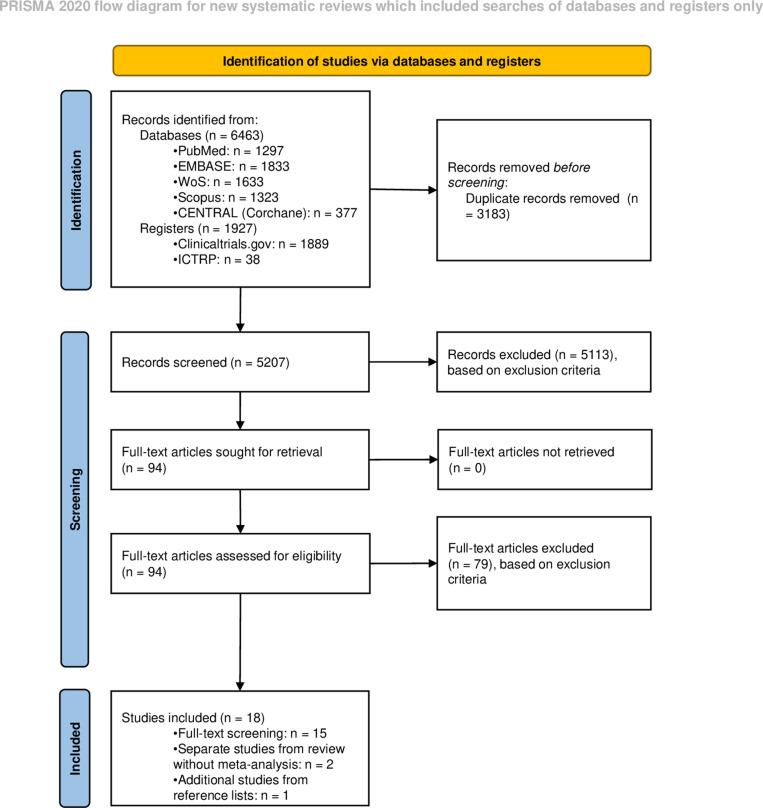
Flow chart of the study selection process. Adapted Preferred Reporting Items for Systematic Reviews (PRISMA) flow diagram indicating search and screening process for the present literature review. *Abbreviations in Alphabetic order: ICTRP =* *International Clinical Trials Registry Platform; n = number; WoS = Web of Science.*

Fig 1 represents the flowchart of the study selection process. Based on the predefined search and eligibility criteria, 17 studies were included. The reference lists of the included studies were screened, and one additional relevant study was identified, resulting in a total of 18 studies included in this review. A detailed overview of the studies can be found in the predefined data extraction chart (S6 File). The majority of the included studies were randomized controlled trials (RCT) (14/18, 78%). The sample size ranged from 14 to 554 participants. It should be noted that of the 18 included studies, the two studies by Thams et al. (2022) were from the same clinical trial, only reporting different outcomes [32,33]. Additionally, the study by Fisch Shvalb et al. (2022) and Yackobovitch-Gavan et al. (2023) also reported the results from the same clinical trial, with Yackobovitch-Gavan et al. (2023) covering the open-label extension phase of the trial [34,35].

### Study populations

The basic study characteristics of the included studies are shown in Table 1. Of the 18 included studies, two studies were conducted in individuals with CP [36,37], one specifically in adolescents and adults with CP [36] and the other in children and adolescents with a brain lesion disorder, including CP [37]. Eight of the included studies were in healthy children [32–35,38–41], two in children with respiratory problems [42,43], one in critically ill children [44], one in children with end-stage liver disease [45], one in children and adolescents undergoing treatment for pediatric malignancy [46], one in children with Pompe disease [47] and two in ambulatory children with Duchenne muscular dystrophy [48,49]. While malnutrition was an exclusion criterion, it should be noted that in the study by Grillenberger et al. (2003), 25% of the enrolled participants had stunting [38].

**Table 1:** Basic study characteristics of the included studies.

Study	Study design	Population	Age range (years)	Number of participants	Intervention	Control	Duration	Muscle outcome	Significant effect
Theis et al. (2021) [36]	RCT	Spastic cerebral palsy	12–25	24	**L-Leucine** 192 mg/kg/d	Placebo	10 weeks	MV elbow flexors	Yes
Yoon et al. (2022) [37]	Observational analytic	Brain lesion disorder	8–19	90	**Protein drink** 6-12g animal protein 4-8g polant protein 1-2g leucine Vitamin D and calcium	NA	12 weeks	MM, FFM	No
Thams et al. (2022) [32]	RCT	Healthy	6-8	200	**High protein yoghurt and vitamin D** 23.4-28.6g protein	Normal-protein yoghurt and placebo tablets	24 weeks	FFM, FFMI	No
Thams et al. (2022) [33]	RCT	Healthy	6-8	200	**High protein yoghurt and vitamin D** 23.4-28.6g protein	Normal-protein yoghurt and placebo tablets	24 weeks	LBM	No
Grillenberger et al. (2003) [38]	RCT	Healthy	7.1^a^	554	**Meal enriched with either meat, milk or extra fat**	No supplement	18 months	MUAC	Yes
Kasture et al. (2023) [39]	RCT	Healthy	6-11	232	**Protein-rich snack and yoga or physical exercise** 7g protein	**Protein-rich snack without yoga or exercise** 7g protein	6 months	FFM	No
Clinicaltrials.gov (2019) [40]	RCT	Healthy, with BMI <+1SD to > -2SD	3-5.4	528	**Protein powder and micronutrients** RDI of Protein	Control beverage powder	6 months	MUAC, AMA	NA
Han et al. (2011) [41]	RCT	Healthy, short stature	7-11	20	**Nutritional supplement** 7 g protein	No nutritional supplement	18 months	LBM	No
Fisch Shvalb et al. (2022) [35]	RCT	Healthy, short and lean prepubertal	10-14.5	158	**Nutritional formula** 36g whey protein	Placebo	6 months	MM, FFM	Yes
Yackobovitch-Gavan et al. (2023) [34]	Observational analytic	Healthy, short and lean prepubertal	10-14.5	98	**Nutritional formula** 36g whey protein	NA	6 months	MM	Yes
Nambi et al. (2022) [42]	RCT	4-week post-COVID-19 obese	5-12	76	**High-protein diet and physical exercise** 1.1-1.2g/kg/d protein	Regular physical activities and dietary patterns	8 weeks	M-CSA arm, thigh and calf	Yes
Martinez et al. (2015) [43]	Observational analytic	Respiratory Insufficiency	0.08-17	20	**Modified diet with adjusted protein intake**	NA	12 weeks	LBM%	No
Hauschild et al. (2019) [44]	RCT	Critically ill admitted at the PICU	0.08-14	25	**Nutritional formula with whey protein** RDI of Protein	Standard protocol	Variable	MUAC	No
Chin et al. (1992) [45]	RCT	End-stage liver disease	0.5-6.75	19	**BCAA enriched formula** 148% of BCAA content	Formula without BCAA enrichment	56 days	MUAC	Yes
Ward et al. (2009) [46]	Observational analytic	Pediatric malignancy	2-21	76	**L-Glutamine** 0.65 g/kg	No supplement	7 days	MUAC	No
Scheffers et al. (2023) [47]	RCT	Pompe disease	6-18	14	**High-protein diet and physical exercise** Total intake of 2g/kg/d	Regular physical activities and dietary patterns	12 weeks	FFM	No
Mok et al. (2009) [48]	RCT	Duchenne muscular dystrophy	2-10	30	**L-Glutamine** 0.5 g/kg/d	Placebo	9 months	FFM	Yes
Davidson et al. (2021) [49]	RCT	Duchenne muscular dystrophy	5-13	36	**Nutritional formula** 0.6g/kg/d glutamine 38mg/kg/d HMB	**Nutritional formula** 22g whey protein Multivitamin Vitamin D Fish oil	42 weeks	LBM, LBM%	No

Abbreviations *in alphabetic order: AMA = Arm Muscle Area; BCAA = Branched-chain amino acid; BW = Body Weight; d = day; FFM = Fat Free Mass; FFMI = Fat Free Mass Index; g = gram; HMB=β-hydroxy-β-methylbutyrate; kg = kilogram; LBM = Lean Body Mass; LBM% = Lean Body Mass percentage; M-CSA = Muscle-Cross-sectional Area; mg = milligram; MM = Muscle Mass; MUAC = Mid-Upper Arm Circumference; MV = Muscle Volume; NA = Not Applicable: RCT = Randomized Controlled Trial; SD = Standard Deviation; μg = microgram; % = percentage.*

^*a*^ *No age range was reported, the median age of the participants was 7.1 years. All children were enrolled in class 1.*

### Effect of protein supplements on muscle outcomes

Different muscle parameters were reported, including elbow flexor muscle volume, skeletal muscle mass, fat-free mass and fat-free mass index, lean body mass and lean body mass percentage, arm muscle area, and muscle cross-sectional area of the arm, thigh and calf. To compare the effects of the different studies on muscle outcomes, the protein amount per kilogram of body weight per day and the estimated equivalent amount of leucine intake based on the type of protein used, were calculated for each specific intervention, as shown in the supporting information (S7 File).

In CP patients, the study by Theis et al. (2021) showed a significant (p < 0.001) increase in elbow flexor muscle volume after 10 weeks of L-leucine supplementation compared to the control group [36]. Conversely, the study by Yoon et al. (2022) reported no significant (p-value not reported) change in skeletal muscle mass or fat-free mass after 12 weeks of protein nutrient drink supplementation [37]. However, it should be mentioned that the amount of leucine equivalent per kilogram of body weight per day provided in the study by Yoon et al. (2022) was less than 50% of the amount of leucine equivalent per kilogram of body weight per day used in the study by Theis et al. (2021) [36,37]. Of the 16 studies conducted in other populations, six studies showed a significant effect on muscle outcomes after protein or amino acid supplementation [34,35,38,42,45,48], while the remaining ten studies did not [32,33,41,39,43,44,46,47,49] or did not report statistics regarding significance [40]. In healthy children, the study by Thams et al. (2022) showed no significant effect on fat-free mass index (p = 0.93) or lean mass (p = 0.42) after 24 weeks of supplementation with high-protein yoghurt and vitamin D tablets [32,33]. Kasture et al. (2023) also failed to demonstrated a significant effect on fat-free mass percentage (p > 0.05) after six months of supplementation with a high-protein snack, combined with yoga or physical exercise [39]. However, it should be noted that the high-protein snack was administered to all study participants and only the level of activity differed between the control and intervention groups. In addition, the mean total protein intake per day did not increase within the expected range based on the use of the protein snack [39]. The RCT conducted by Han et al. (2011), in boys with short stature, also showed a comparable lean body mass between the intervention and control groups after six months of nutritional supplementation (enriched in calories and protein) (p = 0.73) and an additional 12 months of nutritional supplementation combined with growth hormone (p = 0.67) [41]. The RCT by Grillenberger et al. (2003) reported a significant effect (p = 0.019–0.034) on mid-upper arm circumference in Kenyan schoolchildren who consumed an isocaloric food supplement containing either meat, milk, or extra fat for 18 months, with the largest effect observed in the meat group. It should be noted that no details were available on the amount of meat or milk added to the meal [38]. Therefore, the protein amount or leucine equivalents could not be calculated. The study by Fisch Shvalb et al. (2022) also showed a significant effect on fat free mass (p = 0.021) and muscle mass (p = 0.009) after six months of consuming a nutritional supplementation formula (enriched in calories and whey protein) [35], which was continued during the open-label extension phase of the study, reported by Yackobovitch-Gavan et al. (2023) [34]. The clinical trial registration reported change from baseline to six months in mid-upper arm circumference and arm muscle area after the use of a protein powder with micronutrients, but information on protein content and tests for significance were not reported [40]. In children with respiratory insufficiency, Martinez et al. (2015) reported no significant effect (p = 0.12) on mean lean body mass percentage after 12 weeks of a modified diet with adjusted energy and protein intake [43]. It should be noted that the intervention was variable among a study population of only 20 children (with 16 children who completed the study), with some individuals receiving almost no supplemental protein [43]. The study by Nambi et al. (2022) investigated the effect of an eight week intervention with a high-protein diet combined with high-intensity aerobic training in obese male children 4-week post-COVID-19 and showed a significant difference (p < 0.001) in muscle cross-sectional area (arm, thigh, and calf) between the intervention and control groups at baseline, at 8 weeks, and 6 months [42]. Hauschild et al. (2019) reported no statistical difference (p-value not reported) in mid-upper arm circumference after administration of a polymeric or oligomeric whey protein module in critically ill children [44]. Ward et al. (2009) also reported no statistical difference (p = 0.254) in mid-upper-arm circumference after glutamine supplementation in children and adolescents undergoing treatment for pediatric malignancies [46]. It should be noted that the primary goal of the study was to investigate the effect of glutamine on the development of mucositis. Mid-upper arm circumference was assessed as a secondary outcome during the clinical examination, not interpreted as a muscle property [46]. The RCT conducted by Scheffers et al. (2023) in children with Pompe disease showed no significant difference (p = 0.328) in fat-free mass percentage after 12 weeks of a high-protein diet combined with physical exercise [47]. It should be noted that the proposed increase in average protein intake of 2g/kg/day was not achieved by the participants [47]. The study by Chin et al. (1992) did show a significant increase (p < 0.05) in mid-upper arm circumference after nutritional supplementation with a branched-chain amino acid enriched formula in children with end-stage liver disease [45]. Mok et al. (2009) and Davidson et al. (2021) both investigated the effect of nutritional supplementation on muscle outcomes in ambulatory children with Duchenne muscular dystrophy [48,49]. The study by Mok et al. (2009) showed a significant increase (p < 0.0001) in fat free mass after the nine month trial, including four months of glutamine supplementation, after which the effect was attenuated [48]. The study by Davidson et al. (2021) showed no clear changes in lean mass (p = 0.33) and lean mass percentage (p = 0.18) after supplementation with an HMB-enriched protein and vitamin supplement [49]. However, both the control group as well as the treatment group received 22 grams of whey protein in their supplement [49].

### Adverse events and safety issues

Only two studies, both performed in healthy, short and lean prepubertal boys, reported adverse events related to the use of the protein or amino-acid supplement [34,35], and these included mild gastrointestinal symptoms such as sporadic stomach ache, nausea, constipation and “feeling full”. These were only reported by a small number of participants, and no serious adverse events occurred during the studies [34,35]. The other included studies did not report adverse events related to the use of the protein or amino-acid supplement [32,33,40,39,43–49], or did not investigate adverse events [36–38,41,42].

### Critical appraisal

The critical appraisal of the included studies, based on the Downs and Black Checklist for Quality Assessment, is presented in the supporting information (S8 File). Out of the 18 studies included in this review, none were rated as excellent. Eight studies were rated as good [32–36,47–49], nine as fair [38–46] and one as poor [37] in terms of quality. Especially on the items related to external and internal validity, the included studies did not score well.

## Discussion

To our knowledge, our study is the first to review the use of protein supplements and the effect on muscle properties in children with CP. Due to the limited number of studies, a broader search on the use of protein supplements in a pediatric population was conducted. This strategy made it possible to retrieve valuable information on for example possible adverse events. Additionally, it should be noted that hampered or abnormal muscle characteristics can also be present in the other pathologies described. Therefore, it was considered relevant to include these studies. This review included a total of 18 studies, with only two performed in the CP population, making it difficult to draw solid conclusion for this specific population. The studies conducted in other populations used different types of supplements and dosages, which were consumed during variable time periods. The high variability in the used protocols of these included studies, resulted in a large heterogeneity. Based on the critical appraisal, the quality level of the included studies was rather moderate. Therefore, results should be interpreted with caution. Additionally, it should be taken into account that there was a large drop-out during the different clinical trials, ranging from 0 to 165 participants not completing the entire follow-up [40,41,47,48].

No clear statement can be formulated regarding the first research question:’Which protein supplements are used in a pediatric population with as aim increasing muscle volume?’. However, it is interesting to note that studies that used higher amounts of leucine equivalents, through supplementation with high doses of either leucine, BCAA, or whey protein, tended to show a significant effect on muscle parameters [34–36,42,45], while studies that used lower amounts of leucine equivalents did not [37,41,39,43,44,47]. It is generally accepted that a positive net protein balance is necessary for muscle protein synthesis and thus muscle growth [50]. Rapamycin complex 1 (mTORC1) plays a crucial role in postprandial muscle protein synthesis by initiating mRNA translation and reducing protein degradation. Amino acids are essential for the activation of mTORC1. Hereby, the BCAA, and especially leucine, appear to be important, since leucine alone enhances muscle protein synthesis to a similar extent as a complete protein or amino acid mixture [51]. It is therefore tempting to suggest that high doses of leucine or leucine-rich protein supplements might improve muscle protein synthesis in children. However, it should be noted that several confounding factors were not accounted for. For example, none of the studies controlled for the timing of supplementation, and only three studies combined the use of protein supplements with exercise [39,42,47]. Two of the three studies did not show a significant effect on fat-free mass after a high-protein diet combined with physical exercise [39,47]. The study by Nambi et al. (2022), combining a high-protein diet with high-intensity aerobic training and strength training, did show a significant difference in muscle cross-sectional area between the intervention and control groups [42]. However, the increase in protein intake and calculated leucine equivalents in the studies by Kasura et al. (2023) and Scheffers et al. (2023) was much smaller than in the study by Nambi et al. (2022), making it difficult to draw conclusions about the combined effect of protein supplementation and exercise on muscle parameters [39,42,47]. Studies out of the scope of this review, including in healthy adults and in elderly, suggested that protein supplementation on top of resistance training is recommended to improve muscle parameters, including muscle mass [17,18,52]. Therefore, this strategy could be considered to improve muscle growth in children with CP. Timing of protein supplementation, whether in combination with exercise or not, could have a relevant impact on results. For example, supplement intake shortly before or after resistance training showed better results on muscle hypertrophy than supplement intake during hours not in vicinity of resistance training [53]. It is important to note that muscle parameters are sensitive to natural growth, meaning that since children grow over time, muscle parameters will increase as well. In a RCT design, it is expected that this takes place in both the control and intervention group, an therefore does not create a problem. However, in study designs without a control group, natural growth should be taken into account, e.g., via normalization techniques, to counter for expected natural growth in body size. The four studies without an RCT design included in this review, did not report such corrections [34,37,43,46].

Three out of the 18 studies used the non-essential amino acid glutamine as a supplement [46,48,49]. Given the heterogeneity of the studies, no clear statement can be made on the effect of glutamine on muscle properties. From a theoretical perspective, it is puzzling how supplementation with a non-essential amino acid could promote muscle protein synthesis. In animal models, glutamine upregulates mTORC1 mainly in the context of muscle degeneration or inflammation [54]. In fact, cellular leucine uptake is highly dependent on intracellular glutamine concentrations. In conditions of high inflammation, such as sepsis, glutamine is released from skeletal muscle, inhibiting leucine uptake and thus muscle protein synthesis, a phenomenon that may underlie anabolic resistance. Intramuscular glutamine concentration has also been demonstrated to be decreased in Duchenne muscular dystrophy, but the underlying mechanism appears to be related to metabolic rewiring rather than inflammation [55,56]. Taken together, it is prudent to say that studying glutamine supplementation to induce muscle hypertrophy may only be useful in certain populations.

Regarding the second research question: ‘What is known from the existing literature about the benefits and adverse effects of protein supplementation on muscle hypertrophy in children with CP?’, the literature was also limited, with only two studies identified [36,37]. No information on possible adverse effects were reported in either study [36,37]. In general, this review highlights that more research is needed to understand the possible beneficial effects of protein or amino acid supplementation on macroscopic muscle parameters in (children with) CP. As some individuals with CP have nutritional problems, it is important to note that supplementation should not be confused with correcting a malnourished state in which muscle hypertrophy can never be achieved. A better understanding of the pathophysiological mechanisms underlying progressive musculoskeletal dysfunction in individuals with CP will be necessary to guide further therapeutic strategies. Indeed, if mechanisms such as anabolic resistance or inflammation were to contribute, a higher protein requirement per age group (and perhaps glutamine) would be necessary to achieve a positive net protein balance and thus muscle protein synthesis.

Although the focus of this review is on the use of protein supplements, many other nutritional supplements, such as creatine and vitamin D, have been tested in several populations to improve muscle health. Creatine is believed to enhance high-intensity exercise through its conversion to phosphocreatine, which is involved in muscle Adenosine triphosphate regeneration. In a recent meta-analysis in healthy adults, the magnitude of the effect was rather modest, questioning the practical relevance of creatine supplementation [57]. Only one of the studies included in this review used a multicomponent supplement containing creatine, glutamine and a metabolite of leucine in patients with Duchenne muscular dystrophy. No significant changes in muscle parameters were observed. Although vitamin D is primarily involved in bone metabolism, exogenous vitamin D upregulates the expression of the vitamin D receptor in skeletal muscle, increasing anabolic signaling and muscle protein synthesis. Three of the included studies combined protein supplementation with vitamin D [32,33,37], but none of them reached significance regarding muscle parameters.

## Conclusion

This scoping review aimed to examine the extent, range and nature of the available research on the use of protein supplements in children with CP to improve macroscopic muscle characteristics. The limited number of studies conducted on this topic in this specific population, the heterogeneity of the included studies conducted in other pediatric populations, and the lack of high quality of the included studies make it difficult to draw solid conclusions. More research is needed on the use of protein supplements in CP. However, supplementation with BCAA, and more specifically leucine, might be promising.

## Supporting information

S1 File The Preferred Reporting Items for Systematic Reviews and Meta-analysis Protocols (PRISMA-P) checklist, used to review the protocol of the scoping review. (PDF)

S2 File The Preferred Reporting Items for Systematic Reviews and Meta-analysis extension for Scoping Reviews (PRISMA-ScR) checklist, used to review the protocol of the scoping review. (PDF)

S3 File The Peer Review of Electronic Search Strategies (PRESS) checklist, used to review the search strategy of the scoping review. (PDF)

S4 File An extension to the PRISMA Statement for reporting literature searches in systematic reviews (PRISMA-S checklist), used to review the search strategy of the scoping review. (PDF)

S5 File The detailed search strategy for each database, searched during the scoping review. (DOCX)

S6 File Data extraction chart, providing a detailed overview of the studies included in the scoping review. (XLSX)

S7 File Details on the protein and leucine amount used in the different included studies. (DOCX)

S8 File The critical appraisal of the included studies in the scoping review, based on the Downs and Black Checklist for Quality Assessment. (DOCX)

S9 File Final protocol. (PDF)

S10 File The Preferred Reporting Items for Systematic Reviews and Meta-analysis extension for Scoping Reviews (PRISMA-ScR) checklist, used to review the manuscript of the scoping review. (PDF)
