# Supplementary material for: The use of protein supplements in children with cerebral palsy: A scoping literature review
Source: PLoS One. 2025 May 8;20(5):e0322730. doi: 10.1371/journal.pone.0322730 (PMC12061159; doi:10.1371/journal.pone.0322730)
Supplement: S3 File — (PDF) [file pone.0322730.s003.pdf]

S3 File: The Peer Review of Electronic Search Strategies (PRESS) checklist, used to review the search strategy of the scoping review.

Translation of the research question	NA	• Does the search strategy match the research question/PICO?
	YES	• Are the search concepts clear?
	NA	• Are there too many or too few PICO elements included?
	NO	• Are the search concepts too narrow or too broad?
	NA	• Does the search retrieve too many or too few records? (Please show number of hits per line.)
	YES	• Are unconventional or complex strategies explained?
Boolean and proximity operators (these vary based on search service)	YES	• Are Boolean or proximity operators used correctly?
	YES	• Is the use of nesting with brackets appropriate and effective for the search?
	NO	• If NOT is used, is this likely to result in any unintended exclusions?
	NO	• Could precision be improved by using proximity operators (eg, adjacent, near, within) or phrase searching instead of AND?
	YES	• Is the width of proximity operators suitable (eg, might adj5 pick up more variants than adj2)?
Subject headings (database specific)	YES	• Are the subject headings relevant?
	NO	• Are any relevant subject headings missing; for example, previous index terms?
	NO	• Are any subject headings too broad or too narrow?
	NA	• Are subject headings exploded where necessary and vice versa?
	NA	• Are major headings (''starring'' or restrict to focus) used? If so, is there adequate justification?
	NO	• Are subheadings missing?
	NA	• Are subheadings attached to subject headings? (Floating subheadings may be preferred.)
	NA	• Are floating subheadings relevant and used appropriately?
	YES	• Are both subject headings and terms in free text (see the following) used for each concept?
Text word searching (free text)	YES	• Does the search include all spelling variants in free text (eg, UK vs. US spelling)?
	YES	• Does the search include all synonyms or antonyms (eg, opposites)?
	YES	• Does the search capture relevant truncation (ie, is truncation at the correct place)?
	X	• Is the truncation too broad or too narrow?
	YES	• Are acronyms or abbreviations used appropriately? Do they capture irrelevant material? Are the full terms also included?
	OK	• Are the keywords specific enough or too broad? Are too many or too few keywords used? Are stop words used?
	YES	• Have the appropriate fields been searched; for example, is the choice of the text word fields (.tw.) or all fields (.af.) appropriate? Are there any other fields to be included or excluded
	NO	(database specific)?
	NO	• Should any long strings be broken into several shorter search statements?
Spelling, syntax, and line numbers	NO	• Are there any spelling errors?
	NO	• Are there any errors in system syntax; for example, the use of a truncation symbol from a different search interface?
	NO	• Are there incorrect line combinations or orphan lines (ie, lines that are not referred to in the final summation that could indicate an error in an AND or OR statement)?
Limits and filters	YES	• Are all limits and filters used appropriately and are they relevant given the research question?
	YES	• Are all limits and filters used appropriately and are they relevant for the database?
	NO	• Are any potentially helpful limits or filters missing? Are the limits or filters too broad or too narrow? Can any limits or filters be added or taken away?
	YES	• Are sources cited for the filters used?

Abbreviation: PICO, population/problem, intervention/exposure, comparison, outcome.

From reference [6]. © 2015 CADTH. Reprinted with permission.
