# Supplementary material for: The use of protein supplements in children with cerebral palsy: A scoping literature review
Source: PLoS One. 2025 May 8;20(5):e0322730. doi: 10.1371/journal.pone.0322730 (PMC12061159; doi:10.1371/journal.pone.0322730)
Supplement: S5 File — (DOCX) [file pone.0322730.s005.docx]

**S5 File**: **The detailed search strategy for each database, searched during the scoping review.**

1. **Information sources and methods**

| **Name Database** | **Site platform** |
| --- | --- |
| PubMed | https://pubmed.ncbi.nlm.nih.gov/ |
| EMBASE | https://www.embase.com/search/quick |
| Web of Science core collection | https://www.webofscience.com/wos/woscc/basic-search |
| Scopus | https://www.scopus.com/search/form.uri?display=basic#basic |
| CENTRAL (Corchane) | https://www.cochranelibrary.com/ |
| Clinicaltrials.gov | <https://clinicaltrials.gov/> |
| ICTRP | <https://apps.who.int/trialsearch/> |

Cited references were examined by browsing reference lists.

If necessary, additional studies or data was sought by contacting authors.

No online or printed resources (e.g., tables of contents, print conference proceedings, web sites) were browsed.

1. **Search strategy**
   1. **Defining concepts and final full search strategy**

For the search strategy, four concepts were defined based on the objectives of the scoping review. The four concepts are ‘cerebral palsy’, ‘children’, ‘protein supplements’ and ‘muscle volume’. Both the concepts ‘cerebral palsy’ and ‘children’ define the target population. The concept ‘protein supplements’ covers the intervention and the concept ‘muscle volume’ covers the outcome. The outcome concept was only combined with the population concept ‘children’, to limit this broad target population without limiting the narrow population concept ‘cerebral palsy’.

- 1. **Search strategy per database and study register**

**PubMed (including Medline)**

Concept 1: cerebral palsy

"Cerebral Palsy"[Mesh] OR “celebral pals*”[tiab] OR “spastic diplegia*”[tiab] OR “spastic hemiplegia*”[tiab] OR “diplegia spastica”[tiab] OR “cerebral paralys*”[tiab] OR “cerebral pares*”[tiab]

Concept 2: children

"Child"[Mesh] OR “child*”[tiab] OR “paediatric*”[tiab] OR “pediatric*”[tiab] OR “minor”[tiab] OR “minors”[tiab] OR “boy”[tiab] OR “boys”[tiab] OR “girl”[tiab] OR “girls”[tiab] OR “toddler*”[tiab] OR “preschool*”[tiab] OR “pre-school*”[tiab]

Concept 3: protein supplements

"Dietary Supplements"[Mesh:NoExp] OR “dietary supplement*”[tiab] OR “diet supplement*”[tiab] OR “diet additive*”[tiab] OR “dietary additive*”[tiab] OR ((“protein*”[tiab] OR “amino acid*”[tiab] OR "Amino Acids, Branched-Chain"[Mesh] OR "Amino Acids, Essential"[Mesh] OR “leucine”[tiab] OR “isoleucine”[tiab] OR “valine”[tiab] OR “creatine”[tiab]) AND (“supplement*”[tiab] OR “additive*”[tiab] OR “nutrition*”[tiab] OR “diet*”[tiab]))

Concept 4: Muscle volume

"Muscle, Skeletal"[Mesh] OR “muscle”[tiab] OR “muscles”[tiab] OR “muscular”[tiab] OR “cross-sectional area”[tiab]

Final full search strategy= (Concept 1 OR (Concept 2 AND Concept 4)) AND Concept 3

**Embase**

Concept 1: cerebral palsy

'cerebral palsy'/exp OR ‘cerebral pals*’:ti,ab,kw OR ‘spastic diplegia*’:ti,ab,kw OR ‘spastic hemiplegia*’:ti,ab,kw OR ‘diplegia spastica’:ti,ab,kw OR ‘cerebral paralys*’:ti,ab,kw OR ‘cerebral pares*’:ti,ab,kw

Concept 2: children

'child'/exp OR ‘child*’:ti,ab,kw OR ‘paediatric*’:ti,ab,kw OR ‘pediatric*’:ti,ab,kw OR ‘minor’:ti,ab,kw OR ‘minors’:ti,ab,kw OR ‘boy’:ti,ab,kw OR ‘boys’:ti,ab,kw OR ‘girl’:ti,ab,kw OR ‘girls’:ti,ab,kw OR ‘toddler*’:ti,ab,kw OR ‘preschool*’:ti,ab,kw OR ‘pre-school*’:ti,ab,kw

Concept 3: protein supplements

'protein supplementation'/exp OR 'dietary supplement'/exp OR 'dietary supplement*':ti,ab,kw OR ‘diet supplement*’:ti,ab,kw OR ‘diet additive*’:ti,ab,kw OR ‘dietary additive*’:ti,ab,kw OR ((‘protein*’:ti,ab,kw OR ‘amino acid*’:ti,ab,kw OR 'essential amino acid'/exp OR 'branched chain amino acid'/exp OR ‘leucine’:ti,ab,kw OR ‘isoleucine’:ti,ab,kw OR ‘valine’:ti,ab,kw OR ‘creatine’:ti,ab,kw) AND (‘supplement*’:ti,ab,kw OR ‘additive*’:ti,ab,kw OR ‘nutrition*’:ti,ab,kw OR ‘diet*’:ti,ab,kw))

Concept 4: Muscle volume

'skeletal muscle'/exp OR ‘muscle’:ti,ab,kw OR ‘muscles’:ti,ab,kw OR ‘muscular’:ti,ab,kw OR ‘cross-sectional area’:ti,ab,kw

Final full search strategy= (Concept 1 OR (Concept 2 AND Concept 4)) AND Concept 3 NOT ‘conference abstract’:it

**Web of Science Core Collection**

Concept 1: cerebral palsy

TS=(“cerebral pals*” OR “spastic diplegia*” OR “spastic hemiplegia*” OR “diplegia spastica” OR “cerebral paralys*” OR “cerebral pares*”)

Concept 2: children

TS=(“child*” OR “paediatric*” OR “pediatric*” OR “minor” OR “minors” OR “boy” OR “boys” OR “girl” OR “girls” OR “toddler*” OR “preschool*” OR “pre-school*”)

Concept 3: protein supplements

TS=(“protein supplement*” OR “dietary supplement*” OR “diet supplement*” OR “diet additive*” OR “dietary additive*” OR ((“protein*” OR “amino acid*” OR “leucine” OR “isoleucine” OR “valine” OR “creatine”) AND (“supplement*” OR “additive*” OR “nutrition*” OR “diet*”)))

Concept 4: Muscle volume

TS=(“muscle” OR “muscles” OR “muscular” OR “cross-sectional area”)

Final full search strategy= (Concept 1 OR (Concept 2 AND Concept 4)) AND Concept 3 NOT DT=("meeting abstract”)

**Scopus**

Concept 1: cerebral palsy

TITLE-ABS (“cerebral pals*” OR “spastic diplegia*” OR “spastic hemiplegia*” OR “diplegia spastica” OR “cerebral paralys*” OR “cerebral pares*”) OR AUTHKEY(“cerebral pals*” OR “spastic diplegia*” OR “spastic hemiplegia*” OR “diplegia spastica” OR “cerebral paralys*” OR “cerebral pares*”)

Concept 2: children

TITLE-ABS (“child*” OR “paediatric*” OR “pediatric*” OR “minor” OR “minors” OR “boy” OR “boys” OR “girl” OR “girls” OR “toddler*” OR “preschool*”) OR “pre-school*” OR AUTHKEY(“child*” OR “paediatric*” OR “pediatric*” OR “minor” OR “minors” OR “boy” OR “boys” OR “girl” OR “girls” OR “toddler*” OR “preschool*” OR “pre-school*”)

Concept 3: protein supplements

TITLE-ABS (“protein supplement*” OR “dietary supplement” OR “diet supplement*” OR “diet additive*” OR “dietary additive*” OR ((“protein*” OR “amino acid*” OR “leucine” OR “isoleucine” OR “valine” OR “creatine”) AND (“supplement*” OR “additive*” OR “nutrition*” OR “diet*”))) OR AUTHKEY(“protein supplement*” OR “dietary supplement” OR “diet supplement*” OR “diet additive*” OR “dietary additive*” OR ((“protein*” OR “amino acid*” OR “leucine” OR “isoleucine” OR “valine” OR “creatine”) AND (“supplement*” OR “additive*” OR “nutrition*” OR “diet*”)))

Concept 4: muscle volume

TITLE-ABS (“muscle” OR “muscles” OR “muscular” OR “cross-sectional area”) OR AUTHKEY(“muscle” OR “muscles” OR “muscular” OR “cross-sectional area”)

Final full search strategy= (Concept 1 OR (Concept 2 AND Concept 4)) AND Concept 3

**Cochrane Libary (CENTRAL)**

Concept 1: cerebral palsy

#1: [mh "Cerebral Palsy"]

#2: ((celebral NEXT pals*) OR (spastic NEXT diplegia*) OR (spastic NEXT hemiplegia*) OR “diplegia spastica” OR (cerebral NEXT paralys*) OR (cerebral NEXT pares*)):ti,ab,kw

Concept 2: children

#3: [mh "Child"]

#4: (child* OR paediatric* OR pediatric* OR “minor” OR “minors” OR “boy” OR “boys” OR “girl” OR “girls” OR toddler* OR preschool* OR pre-school*):ti,ab,kw

Concept 3: protein supplements

#5: [mh ^"Dietary Supplements"]

#6: ((dietary NEXT supplement*) OR (diet NEXT supplement*) OR (diet NEXT additive*) OR (dietary NEXT additive*) OR ((protein* OR (amino NEXT acid*) OR “leucine” OR “isoleucine” OR “valine” OR “creatine”) AND (supplement* OR additive* OR nutrition* OR diet*))):ti,ab,kw

Concept 4: Muscle volume

#7: [mh "Muscle, Skeletal"]

#8: (“muscle” OR “muscles” OR “muscular” OR “cross-sectional area”):ti,ab,kw

Final full search strategy= (Concept 1 OR (Concept 2 AND Concept 4)) AND Concept 3= ((#1 OR #2) OR ((#3 OR #4) AND (#7 OR #8))) AND (#5 OR #6)

**Clinicaltrials.gov**

Concept 1: cerebral palsy

Concept 2: children

Concept 3: protein supplements

Concept 4: muscle volume

Final full search strategy= (Dietary supplement OR diet additive OR protein OR amino acid OR leucine OR isoleucine OR valine OR creatine) AND (child OR children OR cerebral palsy)

**ICTRP**

Concept 1: cerebral palsy

Concept 2: children

Concept 3: protein supplements

Concept 4: muscle volume

Final full search strategy= (Dietary supplement OR diet additive OR protein OR amino acid OR leucine OR isoleucine OR valine OR creatine) AND (child OR children OR cerebral palsy)

- 1. **Additional information**

During the literature search in PubMed, the restriction ‘no explode’ was used on de search term ‘Dietary Supplements’ since the underlying additional search terms of this MeSH term were not relevant for the current scoping review and were thought to result in noise during the literature search. During the literature search in EMBASE and Web of Science Core Collection, conference abstracts were excluded. This was done due to their limited amount of information and details. During the search for clinical trial records on CLinicaltrials.gov and ICTRP, only clinical trials with results were searched. No further search limitations, restrictions or filters were used. During the literature search in Web of Science Core Collection, all editions (Science Citation Index Expanded (SCI-EXPANDED)--1955-present, Social Sciences Citation Index (SSCI)--1956-present, Arts & Humanities Citation Index (AHCI)--1975-present, Conference Proceedings Citation Index – Science (CPCI-S)--1990-present, Conference Proceedings Citation Index – Social Science & Humanities (CPCI-SSH)--1990-present, Book Citation Index – Science (BKCI-S)--2005-present, Book Citation Index – Social Sciences & Humanities (BKCI-SSH)--2005-present, Emerging Sources Citation Index (ESCI)--2018-present, Current Chemical Reactions (CCR-EXPANDED)--1985-present, Index Chemicus (IC)--1993-present) were searched. During the search in Clinicaltrials.gov, the final full search strategy was entered at the “other terms” field. During the search in ICTRP, the final full search strategy was entered at the search field on the home page. The search strategy as stated above was developed specific for this scoping review, no previous search strategy was reused for this. The search was performed on 02/03/2023 and updated on 13/06/2024.

1. **Peer review**

The development of the research strategy was done with the help of the biomedical reference librarians of the KU Leuven Libraries – 2Bergen – learning Centre Désiré Collen (Leuven, Belgium). The search strategy was peer-reviewed by another expert (K.D.) using the PRESS (Peer Review of Electronic Search Strategies) checklist and the PRISMA-S checklist (An extension to the PRISMA Statement for reporting literature searches in systematic reviews).

1. **Managing records**

**Search results 02/03/2023:**

| **Name Database** | **Total number of records identified** |
| --- | --- |
| After search | |
| PubMed | 1208 |
| EMBASE | 1696 |
| Web of Science core collection | 1512 |
| Scopus | 1231 |
| CENTRAL (Corchane) | 377 |
| Clinicaltrials.gov | 1879 |
| ICTRP | 37 |
| Total = 7940 | |
| After deduplication | |
| Total = 4977 | |
| After screening | |
| Titles and abstracts | 78 (4899 excluded) |
| Full paper | 15 (65 excluded, 2 separate studies included from review without meta-analysis) |
| Total = 15 | |
|  | |
| Cited references | 1 |
| Total number of studies included in review = 16 | |

**Search results 13/06/2024 (only additionally found, screened and added studies mentioned):**

| **Name Database** | **Total number of records identified** |
| --- | --- |
| After search | |
| PubMed | 89 |
| EMBASE | 137 |
| Web of Science core collection | 121 |
| Scopus | 92 |
| CENTRAL (Corchane) | 0 |
| Clinicaltrials.gov | 10 |
| ICTRP | 1 |
| Total = 450 | |
| After deduplication | |
| Total = 230 | |
| After screening | |
| Titles and abstracts | 16 (214 excluded) |
| Full paper | 2 (14 excluded) |
| Total = 2 | |
|  | |
| Cited references | 0 |
| Total number of studies included in review = 2 | |

To deduplicate records from multiple database searches and other information sources, reference manager EndNote Desktop was used. A second check for deduplication was done manually. Screening was done by the two independent researchers (I.V. and D.R.) using the online program Rayyan ([https://rayyan.ai](https://rayyan.ai/)).
