# Supplementary material for: The use of protein supplements in children with cerebral palsy: A scoping literature review
Source: PLoS One. 2025 May 8;20(5):e0322730. doi: 10.1371/journal.pone.0322730 (PMC12061159; doi:10.1371/journal.pone.0322730)
Supplement: S7 File — (DOCX) [file pone.0322730.s007.docx]

**S7 File: Details on the protein and leucine amount used in the different included studies**.

To compare protein and leucine supplemental intake across the included studies, we downloaded JSON files from the FoodData Central website (<https://fdc.nal.usda.gov/>) and extracted in Excel the total protein and total leucine content for each product for which both data points were available. To understand the distribution of leucine content for different types of food, the leucine content of each product was calculated in milligrams of leucine per gram of protein and plotted against the number of grams of protein (Graph 1). Within this broad distribution, planted-based protein sources tended to have an average leucine content of 50 mg of leucine per gram of protein, while meat tended to have an average leucine content of 80 mg of leucine per gram of protein, and dairy products, including whey protein, tended to have an average leucine content of 100 mg of leucine per gram of protein. These averaged amounts were used to calculate the equivalent supplemental leucine intake, expressed in milligrams per kilogram of body weight per day (Table 1). Since the study by Han et al. (2011) used PediaSure (Abbott), for which specific leucine levels have been published, these specific levels (i.e., 92 mg leucine per gram of protein) were used in the calculations. For studies that did not report specific data on supplemental protein intake, equivalent supplemental leucine intake could not be calculated.

Graph 1: Leucine content of each product, calculated in milligrams of leucine per gram of protein, plotted against the number of grams of protein.

Table 1: Equivalent supplemental leucine intake, expressed in milligrams per kilogram of body weight per day.

| **Study** | **Intervention** | **Mean weight (kg)** | **Protein (g/kg/d)** | **Leucine (mg/kg/d)** | **Significant effect** |
| --- | --- | --- | --- | --- | --- |
| Theis et al. (2021)[1] | **L-Leucine**  192 mg/kg/d | 58 | NA | 192 | Yes |
| Yoon et al. (2022)[2] | **Protein drink**  6-12g animal protein  4-8g plant protein  1-2g leucine  Vitamin D and calcium | 8-11yrs: 24-35  12-14yrs: 35-53  15-19yrs: 35-64 | 8-11yrs: 0.28-0.4  12-14yrs: 0.38-0.57  15-19yrs: 0.31-0.57 | 8-11yrs: 48-70  12-14yrs: 63-96  15-19yrs: 52-96 | No |
| Thams et al. (2022)[3] | **High protein yoghurt and vitamin D**  23.4-28.6g protein | 26 | 0.9-1.1 | 90-110 | No |
| Thams et al. (2022)[4] | **High protein yoghurt and vitamin D**  23.4-28.6g protein | 26 | 0.9-1.1 | 90-110 | No |
| Grillenberger et al. (2003)[5] | **Meal enriched with either meat, milk or extra fat** | 20 | NA | NA | Yes |
| Kasture et al. (2023)[6] | **Protein-rich snack and yoga or physical exercise**  7g protein | 23.7-24.7 | 0.28-0.3 | 22-24 | No |
| Clinicaltrials.gov (2019)[7] | **Protein powder and micronutrients**  RDI of Protein | NA | NA | NA | NA |
| Han et al. (2011)[8] | **Nutritional supplement**  7 g protein | 21 | 0.4 | 36.8 | No |
| Fisch Shvalb et al. (2021)[9] | **Nutritional formula**  36g whey protein | 25 | 1.44 | 144 | Yes |
| Yackobovitch-Gavan et al. (2022)[10] | N**utritional formula**  36g whey protein | 25 | 1.44 | 144 | Yes |
| Nambi et al. (2022)[11] | **High-protein diet** **and physical exercise**  1.1-1.2g/kg/d protein | 44 | 1.1-1.2 | 88-120 | Yes |
| Martinez et al. (2015)[12] | **Modified diet with adjusted protein intake** | 32 | 0.5 | 40 | No |
| Hauschild et al. (2018)[13] | **Nutritional formula with whey protein**  RDI of Protein | NA | 0.4 | 40 | No |
| Chin et al. (1992)[14] | **BCAA enriched formula**  148% of BCAA content | NA | NA | 156-186 | Yes |
| Ward et al. (2009)[15] | **L-Glutamine**  0.65 g/kg | NA | NA | NA | No |
| Scheffers et al. (2023)[16] | **High-protein diet and physical exercise**  2 g/kg/d protein | 43.8 | 0.26 | 21 | No |
| Mok et al. (2009)[17] | **L-Glutamine**  0.5 g/kg/d | 19.7 | NA | NA | Yes |
| Davidson et al. (2021)[18] | **Nutritional formula**  0.6g/kg/d glutamine  38mg/kg/d HMB | 32-36.2 | NA | NA | No |

**References**

1. Theis N, Brown MA, Wood P, Waldron M. Leucine Supplementation Increases Muscle Strength and Volume, Reduces Inflammation, and Affects Wellbeing in Adults and Adolescents with Cerebral Palsy. Journal of Nutrition. 2021;151. doi:10.1093/jn/nxaa006

2. Yoon H, Park H su, An X, Park SJ, Go GW, Kim H, et al. Study on the Improvement of Health and Nutrition Status After a 12-week Protein-Rich Supplementation Regimen in Children and Adolescents With Brain Lesions Disorder. Clin Nutr Res. 2022;11. doi:10.7762/cnr.2022.11.1.20

3. Thams L, Stounbjerg NG, Hvid LG, Mølgaard C, Hansen M, Damsgaard CT. Effects of high dairy protein intake and vitamin D supplementation on body composition and cardiometabolic markers in 6-8-y-old children-the D-pro trial. American Journal of Clinical Nutrition. 2022;115. doi:10.1093/ajcn/nqab424

4. Thams L, Hvid LG, Stounbjerg NG, Brønd JC, Mølgaard C, Damsgaard CT, et al. Vitamin D supplementation and increased dairy protein intake do not affect muscle strength or physical function in healthy 6–8-year-old children: the D-pro randomized trial. Eur J Nutr. 2022;61. doi:10.1007/s00394-022-02912-0

5. Grillenberger M, Neumann CG, Murphy SP, Bwibo NO, Van’T Veer P, Hautvast JGAJ, et al. Food Supplements Have a Positive Impact on Weight Gain and the Addition of Animal Source Foods Increases Lean Body Mass of Kenyan Schoolchildren. Journal of Nutrition. 2003. doi:10.1093/jn/133.11.3957s

6. Kasture S, Khadilkar A, Padidela R, Gondhalekar K, Patil R, Khadilkar V. Effect of Yoga or Physical Exercise on Muscle Function in Rural Indian Children: A Randomized Controlled Trial. J Phys Act Health. 2024;21. doi:10.1123/jpah.2023-0182

7. ClinicalTrials.gov [Internet].  Identifier NCT02177942. A Clinical Trial to Study the Impact of a Nutritional Beverage on Tests of Memory in Healthy Preschool Age Children. 2019 [cited 26 Jan 2024]. Available: https://clinicaltrials.gov/study/NCT02177942?term=NCT02177942&rank=1

8. Han JC, Damaso L, Welch S, Balagopal P, Hossain J, Mauras N. Effects of growth hormone and nutritional therapy in boys with constitutional growth delay: A randomized controlled trial. Journal of Pediatrics. 2011;158. doi:10.1016/j.jpeds.2010.09.006

9. Fisch Shvalb N, Lazar L, Demol S, Mouler M, Rachmiel M, Hershkovitz E, et al. Effect of a nutritional supplementation on growth and body composition in short and lean preadolescent boys: A randomised, double-blind, placebo-controlled study. Acta Paediatrica, International Journal of Paediatrics. 2022;111. doi:10.1111/apa.16054

10. Yackobovitch-Gavan M, Lazar L, Demol S, Mouler M, Rachmiel M, Hershkovitz E, et al. The Effect of a Nutritional Supplement on Growth and Body Composition in Short and Lean Preadolescent Boys following One Year of Intervention. Horm Res Paediatr. 2023;96. doi:10.1159/000526671

11. Nambi G, Alghaider M, Elnegamy TE, Basuodan RM, Alwhaibi RM, Vellaiyan A, et al. Clinical (BMI and MRI) and Biochemical (Adiponectin, Leptin, TNF-α, and IL-6) Effects of High-Intensity Aerobic Training with High-Protein Diet in Children with Obesity following COVID-19 Infection. Int J Environ Res Public Health. 2022;19. doi:10.3390/ijerph19127194

12. Martinez EE, Bechard LJ, Smallwood CD, Duggan CP, Graham RJ, Mehta NM. Impact of individualized diet intervention on body composition and respiratory variables in children with respiratory insufficiency: A pilot intervention study. Pediatric Critical Care Medicine. 2015;16. doi:10.1097/PCC.0000000000000428

13. Hauschild DB, Oliveira LDA, Farias MS, Barbosa E, Bresolin NL, Mehta NM, et al. Enteral Protein Supplementation in Critically Ill Children: A Randomized Controlled Pilot and Feasibility Study. Journal of Parenteral and Enteral Nutrition. 2019;43. doi:10.1002/jpen.1416

14. Chin SE, Shepherd RW, Thomas BJ, Cleghorn GJ, Patrick MK, Wilcox JA, et al. Nutritional support in children with end-stage liver disease: A randomized crossover trial of a branched-chain amino acid supplement. American Journal of Clinical Nutrition. 1992;56. doi:10.1093/ajcn/56.1.158

15. Ward E, Smith M, Henderson M, Reid U, Lewis I, Kinsey S, et al. The effect of high-dose enteral glutamine on the incidence and severity of mucositis in paediatric oncology patients. Eur J Clin Nutr. 2009;63. doi:10.1038/sj.ejcn.1602894

16. Scheffers LE, Somers OC, Dulfer K, Dieleman GC, Walet S, van der Giessen LJ, et al. Physical training and high-protein diet improved muscle strength, parent-reported fatigue, and physical quality of life in children with Pompe disease. J Inherit Metab Dis. 2023;46. doi:10.1002/jimd.12607

17. Mok E, Letellier G, Cuisset JM, Denjean A, Gottrand F, Alberti C, et al. Lack of functional benefit with glutamine versus placebo in Duchenne muscular dystrophy: A randomized crossover trial. PLoS One. 2009;4. doi:10.1371/journal.pone.0005448

18. Davidson ZE, Hughes I, Ryan MM, Kornberg AJ, Cairns AG, Jones K, et al. Effect of a multicomponent nutritional supplement on functional outcomes for Duchenne muscular dystrophy: A randomized controlled trial. Clinical Nutrition. 2021;40. doi:10.1016/j.clnu.2021.06.008
