# Supplementary material for: The use of protein supplements in children with cerebral palsy: A scoping literature review
Source: PLoS One. 2025 May 8;20(5):e0322730. doi: 10.1371/journal.pone.0322730 (PMC12061159; doi:10.1371/journal.pone.0322730)
Supplement: S9 File — (PDF) [file pone.0322730.s009.pdf]

# The use of protein supplements in children with cerebral palsy: a scoping literature review

## A scoping review protocol: Updated

Registration to Open Science Framework

*Submission date: 03/04/2024*

**Contact:** Ineke Verreydt

*This protocol for our scoping review is based on the Preferred Reporting Items for Systematic Reviews and Meta-analysis Protocols (PRISMA-P), the Preferred Reporting Items for Systematic Reviews and Meta-analysis extension for Scoping Reviews (PRISMA-ScR) and the Best practice guidance and reporting items for the development of scoping review protocols by Peters et al. (2022).*

**Review title and timescale**

1. Scoping review title
   The use of protein supplements in children with cerebral palsy: a scoping literature review
2. Anticipated start date
   2/1/2023
3. Anticipated completion date
   1/1/2024
4. Stage of the review at time of this submission

| | Not yet started | Started | Completed |
|---|---|---|---|
| Preliminary searches | | | x |
| Piloting of the study selection process | | | x |
| Formal screening of search results against eligibility criteria | | | x |
| Data extraction | | | x |
| Risk of bias (quality) assessment | | | x |
| Data analysis | | | x |

Provide any other relevant information about the stage of the review here: Ready for submission

**Review general information**

5. Type of review
   Scoping review
6. Language
   English
7. Country
   Belgium
8. Registration
   This protocol was registered prospectively with the Open Science Framework on 27/02/2023. An updated version of the protocol was drafted and registered on the basis of newly acquired knowledge while performing screening and data extraction.
9. Other registration details
   Not applicable
10. Reference and/or URL for published protocol
    This review has not been submitted yet.
11. Dissemination plans
    The summary of results will be sent to University hospitals of Leuven and other relevant policy-makers and researchers working with pediatric populations who might benefit from protein supplementation. Via a discussing meeting, for which all relevant stakeholders will be invited, the implications of our scoping review and key messages will be reviewed. In line with the FWO policy,

the results of this scoping review will be published in an open-access journal. Additionally, the networks of our team members will be used to encourage broad distribution of our results.

12. Keywords

    Cerebral palsy, pediatric, protein supplement, muscle volume

13. Details of any existing review of the same topic by the same authors

    Not applicable

14. Current review status

    This scoping review has not been submitted yet.

15. Any additional information

    Not applicable

16. Details of final report/publication(s)

    Not applicable (review not submitted yet)

**Review team details**

17. Name contact person

    Ineke Verreydt

18. Contact email

    ineke.verreydt@kuleuven.be

19. Contact address

    Clinical Motion Analysis Laboratory, Weligerveld 1, 3212 Pellenberg, Belgium

20. Contact phone number

    0032470804594

21. Organizational affiliation of the review

    KU Leuven, Research group for Neurorehabilitation, Tervuursevest 101 – 3000 Leuven, Belgium

22. Review team members and their organizational affiliations

    Give the title, first name and last name of all members of the team working directly on the review. Give the organizational affiliations and contribution of each member of the review team.

| Title | First name | Laste name | affiliation | Contribution |
|---|---|---|---|---|
| Ms | Ineke | Verreydt | KU Leuven, Research group for Neurorehabilitation, Tervuursevest 101 – 3000 Lueven, Belgium | Development of the protocol; Independent researcher 1 |
| Prof. | Daisy | Rymen | KU Leuven/UZ Leuven, Research unit Woman and Child, Department of Development and regenaration, Herestraat 49 – 3000 Leuven, Belgium | Independent researcher 2 |
| Prof. | Kaat | Desloovere | KU Leuven, Research group for Neurorehabilitation, Tervuursevest 101 – 3000 Leuven, Belgium | Guarantor of the review; Independent researcher 3 in case of discussion |

23. Funding sources/sponsors

    FWO Vlaanderen

24. Role of funders/sponsors

    The funders/sponsors had no contribution in conducting the review or in developing the protocol

25. Conflicts of interest

    Authors have no known conflicts of interest to declare.

**Review methods**

26. Rationale

    Despite the frequent use of protein supplements in other populations (healthy adults, athletes, elderly, critical ill), only few studies have been conducted on the use of protein supplements in a pediatric population or more specific in children with cerebral palsy. Therefore, a systematic review with meta-analysis was considered not applicable. To map the key concepts and findings regarding this research topic, performing a scoping review was preferred.

27. Review objective(s)

    The aim of this scoping review is to examine the extend, range and nature of the available research and to summarize this literature regarding the state-of-the-art on the use of protein supplements to increase macroscopic muscle properties, with a main focus on muscle volume, in a pediatric population in general, and more specifically children with cerebral palsy. The specific research questions are:

    - Which protein supplements are used in a pediatric population, with as main goal increasing muscle volume?
    - What is known from the existing literature about the effects and side effects of protein supplements on muscle volume in children with cerebral palsy?

    For a more detailed explanation on the inclusion and exclusion criteria, see the PCC (Population/Concept/Context) items explained below.

28. Condition or domain being studied

    Protein supplementation use to increase macroscopic muscle properties, with a main focus on muscle volume.

29. Participants/Population

    The end goal is to obtain more insight in the use of protein supplements to increase muscle volume in children with cerebral palsy. The broader search on a pediatric population in general (aged between 2 and 12 years, healthy or diagnosed with a condition other than cerebral palsy) and adults with cerebral palsy is considered to be relevant since, to our knowledge, no studies have been performed on the use of protein supplements to increase muscle volume in children with cerebral palsy. Studies including only undernourished or malnourished participants will be excluded.

30. Concept

    a. Intervention(s)/Exposure(s)

       Protein supplementation with as a main goal to influence the macroscopic morphology of the muscle (e.g. volume, cross-sectional area, length). Examples of protein supplements can include, but are not limited to: essential amino acids, branched chain amino acid, leucine, isoleucine, valine and creatine. All periods of time and duration of

follow-up are eligible. Studies only including intravenous or parenteral administration of supplements will be excluded.

b. Comparator(s)/Control(s)

Any comparator or control is relevant for inclusion, such as studies comparing one form of protein supplements to another form or comparison with any form of placebo. However, studies without a comparator are also eligible for inclusion.

c. Primary outcome(s)

The primary outcome of this scoping review is the protein supplement (e.g. type, dose, timing, form of administration) that is applied with as a main goal to influence the macroscopic properties of the muscle (e.g. volume, cross-sectional area, length, echo-intensity). Studies including only microscopic muscle outcomes or outcomes retrieved from blood or urine analyses will be excluded.

d. Secondary outcome(s)

Reported side effects of the protein supplementation.

e. Types of study to be included initially

All types of published articles are thought to be relevant, but case reports and case series are not preferred due to the descriptive nature. These will be handled as such. Reviews will be included in case a meta-analysis was performed. If not, the separate relevant papers mentioned in the review are screened and if matching the inclusion criteria, included.

31. Context

The eligibility criteria context was not included in this scoping review due to the irrelevance in accordance to the current topic.

32. Eligibility criteria

Eligibility criteria were defined a priori and based on the PCC (Population/Concept/Context) framework as clarified in 29 – 31. Only articles written in English will be included. Articles with as a publication status 'Published' will be taken into account, no time criteria is considered.

33. Information sources

Comprehensive literature searches of electronic bibliographic databases will be conducted by two independent researchers (I.V. and D.R.), in Scopus, Web of Science, PubMed (including Medline), EMBASE and Cochrane Library (CENTRAL). The search will use predefined search terms and MeSH terms. To search for registered clinical trials, Clinicaltrials.gov and ICTRP will be searched as well.

34. Search strategy

Title and abstract of articles obtained through the structured search, will be screened based on predefined eligibility criteria, before analyzing the full article. The reference lists of all relevant reviews and included final papers will also be scanned. The development of the research strategy was done with the help of the biomedical reference librarians of the KU Leuven Libraries – 2Bergen – learning Centre Désiré Collen (Leuven, Belgium). The search strategy was peer-reviewed by another expert (K.D.) using the PRESS (Peer Review of Electronic Search Strategies) checklist and the Extension to the PRISMA Statement for reporting Literature Searches in Systematic Reviews (PRISMA-S) checklist. A draft of the search strategy used for one database, namely PubMed, is presented in Appendix A of this protocol. The final full search strategy for each database, including planned limits, will be made available with the publication. The final full search strategy will be reported using Extension to the PRISMA Statement for reporting Literature Searches in Systematic Reviews (PRISMA-S) and the PRISMA 2020 flow diagram for new systematic reviews.

35. URL to search strategy

Not applicable.

36. Data extraction (selection and coding)

The data abstraction will be performed by a pair of researchers (I.V. and D.R.) independently. Each phase of the review (screening, eligibility, inclusion) will be preceded by a training session. Titles and abstracts will be screened using the a priori defined eligibility criteria. Subsequently, full-text articles and citations will be screened for inclusion. Inter-rater discrepancies will be resolved by discussion or a third reviewer (K.D.). We will abstract data on characteristics of the articles (e.g., type of article or study, country of origin, year of publication, first author, last author and if the study was sponsored yes or no), participant characteristics (e.g., population or patient group, age category, gender, number of included participants and presence of a control group yes or no), intervention characteristics (e.g., type of supplements used, duration and dosage of use, timing and form of administration), control characteristics (supplementation), outcome characteristics (e.g., primary outcome parameters, secondary outcome parameters and side effects) and result characteristics (e.g. statistical analysis performed yes or no, summary results muscle outcomes) using a predefined data extraction chart. The standardized data charting form was developed a priori and pilot-tested among all reviewers. To deduplicate records from multiple database searches and other information sources, reference manager EndNote Desktop will be used. A second check for deduplication will be done manually. Screening will be done by the two independent researchers using the online program Rayyan (https://rayyan.ai).

37. Risk of bias (quality) assessment

The Downs and Black Checklist for Quality Assessment, able to evaluate the quality of both randomized controlled and non-controlled trials, was used. Due to the nature of this review, the scoring of item 27 was modified (it was rated whether the study did or did not performed a power calculation, instead of rating according to an available range of study powers), resulting in a maximum possible score of 28, instead of 32. Additionally, a corresponding quality level was given to the included study: excellent (26-28); good (20-25); fair (15-19); and poor (</=14). The scoring was performed by a single reviewer (I.V.), but discussed with the second reviewer (D.R.).

38. Strategy for synthesis of results

The data synthesis has as a main goal to summarize the literature regarding the state-of-the-art of the use of protein supplements to increase muscle volume in a pediatric population in general, and more specifically in children with cerebral palsy. By synthesizing this information, we will identify the most suitable protein supplements for an intervention with nutritional supplements in children with CP, as well as important remarks concerning safety issues and protocol guidelines. This will be achieved by summarizing the literature according to article, participant, intervention, outcome and result characteristics. This will be done based on a data extraction chart, which will be available in the appendix of the publication. If possible, studies and study results will be grouped based on the specific protein supplements that were used. Extracted data will be presented in tables. We will present a general interpretation of the results in relation to the objectives of the study, as well as potential implications and possible next steps. In addition, we will consider qualitative analysis (e.g., content analysis) for open-text data, as necessary, which will be conducted by two reviewers independently.

39. Analysis of subgroups or subsets

Not applicable

## Appendix A: Draft of the search strategy in PubMed

Concept 1: cerebral palsy

"Cerebral Palsy"[Mesh] OR "celebral pals*"[tiab] OR "spastic diplegia*"[tiab] OR "spastic hemiplegia*"[tiab] OR "diplegia spastica"[tiab] OR "cerebral paralys*"[tiab] OR "cerebral pares*"[tiab]

Concept 2: children

"Child"[Mesh] OR "child*"[tiab] OR "paediatric*"[tiab] OR "pediatric*"[tiab] OR "minor"[tiab] OR "minors"[tiab] OR "boy"[tiab] OR "boys"[tiab] OR "girl"[tiab] OR "girls"[tiab] OR "toddler*"[tiab] OR "preschool*"[tiab] OR "pre-school*"[tiab]

Concept 3: protein supplements

"Dietary Supplements"[Mesh:NoExp] OR "dietary supplement*"[tiab] OR "diet supplement*"[tiab] OR "diet additive*"[tiab] OR "dietary additive*"[tiab] OR (("protein*"[tiab] OR "amino acid*"[tiab] OR "Amino Acids, Branched-Chain"[Mesh] OR "Amino Acids, Essential"[Mesh] OR "leucine"[tiab] OR "isoleucine"[tiab] OR "valine"[tiab] OR "creatine"[tiab]) AND ("supplement*"[tiab] OR "additive*"[tiab] OR "nutrition*"[tiab] OR "diet*"[tiab]))

Concept 4: Muscle volume

"Muscle, Skeletal"[Mesh] OR "muscle"[tiab] OR "muscles"[tiab] OR "muscular"[tiab] OR "cross-sectional area"[tiab]

Final full search strategy = (Concept 1 OR (Concept 2 AND Concept 4)) AND Concept 3
